# Surviving sepsis: going beyond the guidelines

**DOI:** 10.1186/2110-5820-1-17

**Published:** 2011-06-07

**Authors:** Paul E Marik

**Affiliations:** 1 Division of Pulmonary and Critical Care Medicine, Eastern Virginia Medical School, Norfolk, VA 23507, USA

## Abstract

The Surviving Sepsis Campaign is a global effort to improve the care of patients with severe sepsis and septic shock. The first Surviving Sepsis Campaign Guidelines were published in 2004 with an updated version published in 2008. These guidelines have been endorsed by many professional organizations throughout the world and come regarded as the standard of care for the management of patients with severe sepsis. Unfortunately, most of the recommendations of these guidelines are not evidence-based. Furthermore, the major components of the 6-hour bundle are based on a single-center study whose validity has been recently under increasing scrutiny. This paper reviews the validity of the Surviving Sepsis Campaign 6-hour bundle and provides a more evidence-based approach to the initial resuscitation of patients with severe sepsis.

## 

Sepsis is among the most common reasons for admission to intensive care units (ICUs) throughout the world. During the past two decades, the incidence of sepsis in the United States has tripled and is now the tenth leading cause of death. In the United States alone, approximately 750,000 cases of sepsis occur each year, at least 225,000 of which are fatal [1,2]. Septic patients are generally hospitalized for extended periods, rarely leaving the ICU before 2-3 weeks. Despite the use of antimicrobial agents and advanced life-support, the case fatality rate for patients with sepsis has remained between 20% and 30% during the past 2 decades [1,2].

The Surviving Sepsis Campaign (SSC) is a global effort to improve the care of patients with severe sepsis and septic shock. The campaign was launched by the *Society of Critical Care Medicine*, the *European Society of Intensive Care Medicine *and the *International Sepsis Forum *in 2002. The first Surviving Sepsis Campaign Guidelines were published in *Critical Care Medicine *in 2004 and included 52 recommendations [3]. Ely Lily and Company and Edwards Life Sciences sponsored the guideline process, raising concerns about the integrity of the guidelines [4]. Furthermore, it appeared that the guideline implementation process was part of the marketing strategy for the Eli Lilly Company. The Surviving Sepsis Campaign Guidelines were updated in 2008, and although free of corporate sponsorship and somewhat broader in scope (85 recommendations), the core recommendation's remained largely unchanged [5].

These core recommendations were principally based on the results of a small, single-center study by Rivers et al. (Early Goal-Directed Therapy [EGDT]) whose validity has been recently under increasing scrutiny [6,7]. It is important to note that the majority of recommendations in both sets of guidelines were based on the lowest level of scientific evidence (Grade E - uncontrolled studies, case series and expert opinion). Barochia and colleagues performed a systemic review of the association between the component therapies of the Surviving Sepsis Campaign 6-hour resuscitation bundle and outcome [8]. These authors concluded that the "*current sepsis bundles may force physicians to provide unproven or even harmful care. As administered and studied to date, only antibiotics meet the stated criteria of proof for bundle inclusion*."

Evidence-based medicine is defined as "the conscientious, explicit, and judicious use of the *best current scientific evidence *in making decisions about the care of individual patients" [9]. The practice of evidence-based medicine means integrating clinical acumen with patients' unique clinical features and the best available external evidence from systematic research. Clinical practice guidelines embrace evidence-based medicine by rigorously distilling the highest level of evidence from the literature in an effort to help physicians to provide the best possible care to their patients. The principle construct of the guideline development process is that they should be evidence-based and not opinion-based, be fully transparent, and that the developers and sponsoring organization(s) should be free of significant conflict of interest. It is abundantly clear that the Surviving Sepsis Campaign Guidelines fail to meet these requirements for guideline validity. Regrettably, these guidelines have been endorsed by such organization as the "Institute of Healthcare Improvement" and the "Joint Commission" and have become regarded as the standard of care in the United States and many European counties. The Australian and New Zealand Intensive Care Society (ANZICS) have, however, questioned the validity of these guidelines. Due to concern that the guideline "package" would inappropriately be adopted by quality improvement programs and organizations, ANZICS have declined to endorse the Surviving Sepsis Campaign Guidelines [10].

It has become increasingly apparent that in many patients there is a long delay in both the recognition of sepsis and the initiation of appropriate therapy. This has been demonstrated to translate into an increased incidence of progressive organ failure and a higher mortality. Kumar and colleagues investigated the relationship between the duration of hypotension before antimicrobial administration in 2,600 patients with sepsis-induced hypotension [11]. They reported that the risk of dying increased progressively with time to receipt of the first dose of antibiotic. The Surviving Sepsis Campaign and EGDT have succeeded in putting the "spotlight" on sepsis and have popularized the concept that the early identification and treatment of sepsis is essential to improve the outcome of this potentially fatal disease. The early identification of sepsis, the early administration of appropriate antibiotics, and early hemodynamic resuscitation remain the cornerstone of the management of patients with sepsis. However, as demonstrated by Barochia and colleagues, the major components of the Surviving Sepsis Campaign 6-hour resuscitation bundle are not evidence-based and should be abandoned [8].

The hemodynamic alterations with sepsis are exceedingly complex and include volume depletion, depressed myocardial function, and altered microvascular flow. These changes are dynamic; it has been reported that patients with preserved ventricular function may progress to develop severely depressed contractility [12]. In addition, with progressive volume loading patients may develop severe tissue edema, which compromises tissue oxygenation. This paradigm dictates that that the hemodynamic profile of each patient be dynamically monitored and that therapeutic interventions may need be modified based on these changes [13]. Furthermore, it is evident that the complexity of these changes defies a simple treatment algorithm. The major elements of the "6-hour resuscitation bundle" of the Surviving Sepsis Campaign Guidelines include fluid resuscitation to achieve a central venous pressure (CVP) of >8 cmH_2_O (or 12 cmH_2_O when on a ventilator) and a central venous oxygen saturation (ScvO_2_) > 70% with the use of blood and inotropic agents. There is increasing recognition that the major elements of this bundle are not supported by scientific evidence [8,10,14,15]. Remarkably, these recommendations are based on a small (n = 263), nonblinded, single-center study (the River's EGDT study) [6], in which the lead author had significant undisclosed conflicts of interest and where the validly of the data has been questioned [7]. Furthermore, it is noteworthy that the reported hospital mortality of the standard therapy group in the River's EGDT study was 46%; this compares to 17% in a recent randomized, controlled study that evaluated the outcomes of the 6-hour resuscitation bundle [16].

During the first hours of severe sepsis, venodilatation, transudation of fluid from the vascular space into the tissues, reduced oral intake, and increased insensible loss combine to produce hypovolemia [17]. Ventricular dysfunction and arteriolar dilation volume depletion contribute to impaired global perfusion and organ function. Treating hypovolemia is the most important component of the early management of severe sepsis. However, once the patient has received an adequate fluid challenge, further fluid challenges may not increase cardiac output and global perfusion [13]. Additional fluid may increase interstitial edema and further comprise the microvascular dysfunction that characterizes severe sepsis. The current paradigm of fluid management in patients with sepsis is one of adequate initial fluid resuscitation followed by conservative late-fluid management. Conservative late-fluid management is defined as even-to-negative fluid balance measured on at least 2 consecutive days during the first 7 days after septic shock onset. In a retrospective cohort study, Murphy and colleagues demonstrated that an approach that combines both adequate initial fluid resuscitation followed by conservative late-fluid management was associated with improved survival [18]. A retrospective analysis of the Vasopressin in Septic Shock Trial (VASST) demonstrated that those patients in the quartile with the largest positive fluid balance at both 12 hours and 72 hours had the highest mortality [19]. Additional studies have demonstrated that those patients who have the largest cumulative fluid balance have an increased mortality [20-22].

The optimal time to initiate vasopressor agents has not been rigorously studied. Many patients with severe sepsis will respond to a 2-L fluid challenge and require little additional hemodynamic support. If despite adequate intravascular filling a mean arterial pressure in excess of 65 mmHg cannot be achieved, then vasoconstrictors must be used [23]. The early use of vasoconstrictors is recommended, because it reduces the incidence of organ failure and may prevent excessive volume overload (conservative late fluid management) [23]. Therefore, we recommend that a vasopressor agent (norepinephrine) be started once the patient has received 2 L of crystalloid (NS) [17,24,25]. In cases of life-threatening hypotension (i.e., diastolic blood pressure < 40 mmHg), treatment with vasopressors must be started immediately and concurrently with fluid resuscitation [23]. Norepinephrine (starting at 0.01 μg/kg/min) should be titrated upwards while fluid resuscitation continues. Figure 1 provides an initial approach to the resuscitation of patients with septic shock; however, it is important to emphasize that these patients require close hemodynamic monitoring with dynamic changes as the hemodynamic course evolves. Ongoing fluid and vasopressor resuscitation should be guided by mean arterial pressure, pulse pressure variation, passive leg-raising maneuvers, urine output, oxygenation as well as cardiac output (determined noninvasively), and extravascular lung water measurement [13,26,27]. Bedside echocardiography is critical to determine left ventricular size and function. The central venous pressure (CVP) does not reflect intravascular volume nor does it predict fluid responsiveness and has no place in the resuscitation of patients with sepsis [13,28,29]. Interestingly, in the VASST study those patients who met the Surviving Sepsis Campaign CVP target had the highest mortality [19]. Although there is scant data to suggest that one vasopressor results in better outcomes than another (norepinephrine, epinephrine, vasopressin) [24,30-32], we favor norepinephrine as the first-line agent followed by dobutamine or epinephrine in patients with poor left ventricular (LV) function and vasopressin (fixed dose of 0.03 u/min) in patients with "preserved" LV function and a low systemic vascular resistance (SVR; Figure 1). In patients with sepsis, norepinephrine increases blood pressure, as well as cardiac output, renal, splanchnic, cerebral blood flow, and microvascular blood flow while minimally increasing heart rate [33,34]. Norepinephrine seems to be the ideal first-line agent for the management of septic shock; additional agents should be considered in patients who remain hypotensive or display evidence of inadequate tissue or organ perfusion despite doses of norepinephrine up to 0.2 μg/kg/min. The second/third-line agents should be chosen based on the patient's hemodynamic profile as determined by ECHO and noninvasive assessment of cardiac output.

**Figure 1:**
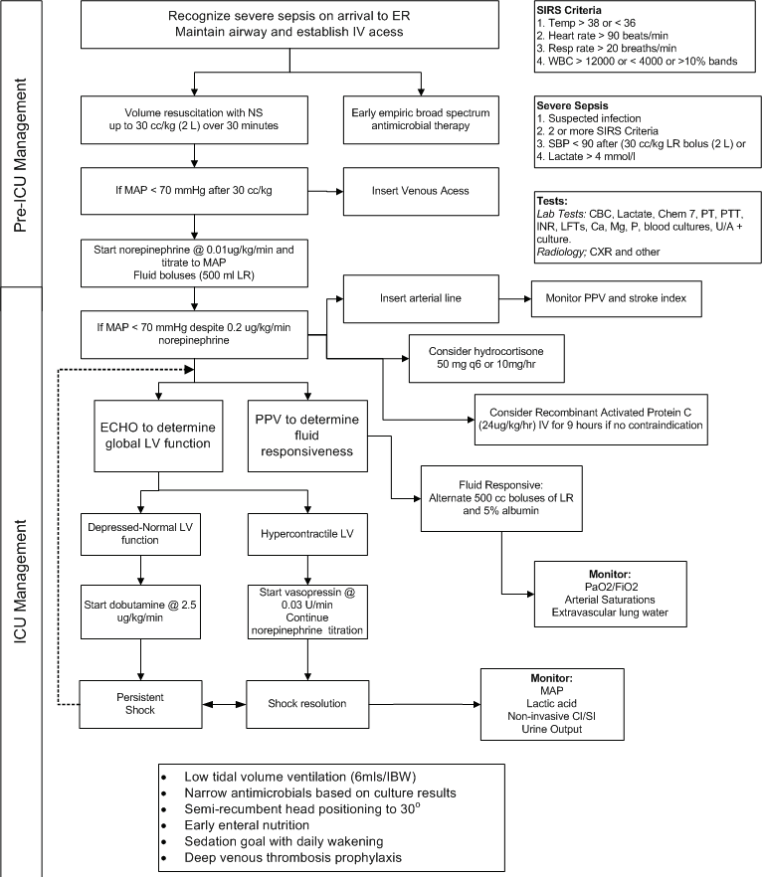
**Suggested initial approach to the management of patients with severe sepsis and septic shock**.

Dopamine has a number of theoretical disadvantages in patients with sepsis. It tends to increase heart rate, increases myocardial oxygen demand, and is associated with splanchnic mucosal ischemia. In addition, dopamine inhibits T and B lymphocytes and decreases secretion of prolactin, growth hormone, and TSH. In a large randomized, controlled trial, De Backer and colleagues compared dopamine with norepinephrine for the treatment of patients with shock [35]. In the subgroup of patients with septic shock, there was a trend toward improved outcome with norepinephrine; however, this difference did not reach statistical significance. A recent meta-analysis that compared norepinephrine to dopamine in patients with septic shock demonstrated a higher mortality with a significantly greater risk of arrhythmias with the use of dopamine [24]. Therefore, this drug should be avoided in patients with sepsis. Similarly phenylephrine is not recommended, because in experimental models it decreases cardiac output as well as renal and splanchnic blood flow [36]. Furthermore, these agents have not been rigorously tested in randomized, controlled studies.

EGDT and the Surviving Sepsis Campaign Guidelines call for the administration of a blood transfusion in patients' with a central venous oxygen saturation (ScvO_2_) of less than 70% and a hematocrit of less than 30% (Grade 2C recommendation) [3,5,6]. This recommendation is a deviation from currently accepted transfusion practices, and as stated by Barochia and colleagues, this recommendation "may force physicians to provide unproven or even harmful care" [8]. In the critically ill, blood transfusions increase the risk of infections, acute respiratory distress syndrome (ARD), multisystem organ failure (MSOF), and death [37-39]. Although the intent of blood transfusions is to increase tissue oxygenation, blood transfusions paradoxically may have the opposite effect. Poorly deformable transfused red blood cells may impede microvascular flow [40]. Furthermore, the P_50 _of stored red cells may be as low as 6 mmHg with the red blood cells being able to unload less than 6% of the carried oxygen; stored cell may thereby increase the ScvO_2 _(by binding oxygen) but compound the tissue oxygen debt by decreasing oxygen unloading.

Central venous oxygen saturation (ScvO_2_) was used as the major endpoint of resuscitation in the intervention arm of the River' EGDT study (CVP > 8 cmH_2_O was targeted in both the control and intervention groups) and is a Grade 2C recommendation in the Surviving Sepsis Campaign Guidelines [5,6]. This is problematic for a number of reasons [29]. Septic patients usually have a normal or increased ScvO_2 _due to reduced oxygen extraction [41,42]. A normal ScvO_2 _therefore does not exclude tissue hypoxia. A low ScvO_2 _is an important sign of inadequate oxygen delivery to meet systemic oxygen demands. However, it provides no information for the reason for this inadequacy, nor does it provide guidance as to the optimal therapeutic approach. It is noteworthy that in the Rivers study the mean ScvO_2 _was 49% with 65% of patients having a ScvO2 less than 70% [6].

No other sepsis study has reproduced this finding; the mean ScvO_2 _(on presentation) in most sepsis studies is approximately 70% [43-45]. This suggests that other factors may have been in play to account for the low ScvO_2 _in the Rivers study [15,46]. These factors include the delayed presentation to hospital (possibly due to socioeconomic factors), greater number of patients with comorbid medical conditions, and a high incidence of alcohol use [15]. Thus, the combination of significant comorbidities (including heart disease) and a more delayed arrival of patients to the emergency department in the River's study may have led to a low cardiac output state, and in turn, to the very low ScvO_2 _values [44]. In a multicenter center EGDT study that enrolled 619 patients, Pope et al. reported that a high ScvO_2 _(ScvO_2 _> 90%) was an independent predictor of death [47]. In this study, a low initial ScvO_2 _(ScvO_2 _< 70%) was not predictive of mortality.

Although aggressive early fluid resuscitation followed by vasoactive agents (in those with persistent hypotension) remains the cornerstone of the management of patients with severe sepsis and septic shock, the endpoints of resuscitation should be based on validated physiologic variables that are individualized based on each patients' comorbidities and unique clinical circumstances [13]. It is unlikely that a "one-size fits all" approach will be appropriate for all patients. A number of multicenter, randomized, controlled studies (ProCESS, ARISE) are currently being undertaken, which are testing the effectiveness of EGDT versus standard therapy [8]. These studies should aid in the development of an evidence-based approach to the early resuscitation of patients with severe sepsis and septic shock.

## Conclusions

The past two decades have seen a remarkable growth in our understanding of sepsis and the complex interconnection of multiple biological pathways involved in the septic process. Despite initial enthusiasm with "disease-modifying agents," the early administration of appropriate antibiotics and early hemodynamic resuscitation remain the cornerstone of the management of patients with sepsis. This resuscitation of patients with sepsis should be based on the best current scientific evidence and coordinated by intensivists with expertise in managing these complex patients. Unfortunately, despite good intentions the Surviving Sepsis Campaign Guidelines are opinion-based rather than evidence-based and should be abandoned. Expert opinions are important but should be labeled as such and not be incorporated into evidence-based clinical practice guidelines.

## Competing interests

The authors declare that they have no competing interests.
